# Pathology for General Practitioners

**Published:** 1907-11-02

**Authors:** 


### Points in Pathology

### PATHOLOGY FOR GENERAL PRACTITIONERS.

### The Apparatus Required for Simple Investigations.

The apparatus required for simple pathological investigations such as can easily be performed by the general practitioner include:—

1. The microscopical examination of pus, sputum, and other materials for micro-organisms.
2. The microscopical examination of stained blood-films. (This does not include a "blood-count," which entails a rather expensive outfit, namely, Thomas's hæmocytometer, costing £1 16s., and Gower's hæmoglobinometer, costing £1 1s.)
3. The testing of blood for Widal's reaction.
4. The cutting and examination of frozen sections.

With regard to (1) it must be understood that the identity of a micro-organism cannot be absolutely determined by means of the microscope; for this it is necessary to inoculate artificial media, to observe the growths obtained, and even to proceed to experiments on animals, all of which can only be carried out in a laboratory. Such cultural experiments are absolutely necessary when the pus contains either a very great number of micro-organisms or so few that it is difficult or impossible to find them with the microscope. Very frequently, however, they are not so essential, as, for instance, when examining for tubercle bacilli or for gonococci (which do not grow readily on artificial media), or when differentiating between staphylo- and streptococci; in these cases the microscope will give almost conclusive evidence as to the nature of the organism. With regard to the diagnosis of diphtheria, it is now generally held that a direct microscopical examination of the exudation in the throat is quite unreliable, and that cultural tests are necessary. The investigation does not, therefore, fall within the scope of the general practitioner.

To consider, now, the apparatus required. The most important, as well as the most expensive, item will be the microscope. Those who are not obliged to consider expense will, of course, get a first-class microscope from a well-known maker, paying any price from £25 upwards. Those, however, who are not so fortunate can get a thoroughly satisfactory instrument at a much lower price, and it must be remembered that a microscope intended merely for diagnosis need not be either so accurate or so elaborate as one intended for research work.

The following are the essential parts of a microscope, and each should be carefully examined before purchasing:—

(*a*) *The Stand.*—This must rest firmly on the foot, which may be of the tripod or of the horseshoe pattern. The Stand should always be capable of inclination from the vertical, and its stability when in this position is to be carefully tested. The Stage should be of sufficient size and have a large aperture; the best covering for the upper surface is vulcanite. The Focussing apparatus is of great importance. A coarse and a fine adjustment are necessary, the former actuated by a rack and pinion, the latter by a micrometer screw. These should be examined with the greatest care, as nothing is more annoying than a faulty adjustment.

(*b*.) *Objectives.*—Two at least will be required, a $\frac{1}{2}$ or $\frac{2}{3}$-in., and a $\frac{1}{6}$-in. In order to examine bacteria a $\frac{1}{12}$-in. oil immersion lens is also necessary. Refined cedar-wood oil is to be used for immersing this objective. The oil may be removed from the lens after use by wiping with a soft handkerchief moistened with saliva; xylol must not be used for this purpose as it has a solvent action on the cement.

(*c*.) *Eye-pieces.*—For ordinary purposes one eye-piece is sufficient. A No. 4 is most generally useful.

(*d*.) *Accessories.*—Some form of Condenser is a necessity for use with the $\frac{1}{12}$-in. objective. Abbé's condenser is cheap and satisfactory. A triple nose-piece to carry the three objectives, though not essential, is of great convenience, and saves much time in changing the objectives.

The "Bacteriological" microscope made by Messrs. Swift,* of Tottenham Court Road, possesses all the parts mentioned above, and is a thoroughly reliable instrument in every way. The price is £15,† including an oil immersion lens. The cost can be lessened by getting a second-hand stand and fitting to it new or even second-hand lenses. Messrs. Clarkson, of 338, High Holborn, make a speciality of second-hand microscopes, and can often supply a stand with Abbé's condenser and triple nose-piece for £5. A new eye-piece will cost 7s. 6d., and new objectives ($\frac{2}{3}$, $\frac{1}{6}$, and $\frac{1}{12}$ in.) about £1, £1 15s., and £5 respectively, bringing the total cost to just over £13. By getting second-hand eye-pieces and objectives this may be still further reduced to about 10 guineas.

Very great care is required in choosing a second-hand microscope, and a little expert advice, if it can be obtained, is invaluable. It is particularly important to see that the lenses of the eye-piece and objectives are not scratched, and that the focussing adjustments work properly. With care, judgment, and, perhaps, a little luck, it is possible to get a very satisfactory second-hand microscope.

There are two common patterns of freezing microtome:—Williams', price £3 10s., and Cathcart's improved pattern, price £1 7s. 6d. The first of these will, of course, give the better results, but very good sections can be cut with the latter. Cathcart's original pattern is not recommended.

It is the truest economy to get the very best stains; in the writer's experience those made by Grübler, of Leipsic, cannot be excelled. They can

* The writer mentions certain firms by name, solely because he happens to be more familiar with their apparatus than with that of other firms. Most of the well-known makers quote very similar prices.

† The prices given in this article are only approximate.

be supplied by most makers of chemical apparatus. Methylene blue, gentian violet, and eosin it is convenient to get in the solid form and to make up into solutions as required. A 10-gram bottle costs from 9d. to 1s., and will give stain sufficient for many month's work. Jenner's blood stain is troublesome to work with; in the first place it is expensive, a one-ounce bottle costing about 1s.; in the second, it deteriorates very rapidly unless the air is carefully excluded. There are on the market many inferior brands of Jenner's stain, which are almost useless. Leishmann's blood stain is a modification of Jenner's stain. The cost and method of use are similar. Hæmatoxylin it is advisable to get as Ehrlich's solution already made up. This may be bought from any chemical dealer. The stain improves greatly on keeping, and is not usually sent out for sale until it has ripened for six months or more. The cost is 5s. 6d. per half pound. The 5 per cent. solution recommended for staining frozen sections can be used over and over again.

Of other reagents only a few need be noticed. Absolute alcohol is very expensive, about 4s. per lb.; whereas methylated spirit only costs 6d. per lb. The amount of absolute alcohol required for dehydrating sections can be lessened by extracting as much water as possible with methylated spirit, when a few drops of alcohol will complete the process. The cost of xylol is 2s. 3d. per lb., of formalin 1s. 6d. per lb.

Coverslips cost from 3s. to 4s. 6d. per oz. One ounce contains about 150 coverslips. Slides, 3 in. by 1 in., cost 2s. 6d. a gross.

Schüster's alkalimeters are small glass bottles shaped like a retort, of a capacity of about 50 c.c. They are very useful for holding alcohol, xylol, and the various staining solutions, price 6d. each. Glass petri dishes are convenient for holding the various washing and staining baths required for the preparation of sections, price 6d. each. Watch glasses may be used for this purpose, but they are awkwardly small. A porcelain tile, from 8 in. to 12 in. square, is generally useful, price 1s.

To clean slides and coverslips boil them in 40 per cent. caustic soda for five minutes, wash well in water, boil in 50 per cent. nitric acid, and again wash. This will effectually remove all the grease. They should then be kept in methylated spirit in covered glass dishes (price 1s.). New slides and coverslips are sometimes quite free from grease, and may then be placed directly in methylated spirit. Slides that have been used need not be thrown away, as they may be cleaned by the above method and used again.

